# Neo-Russellian Abstractionism

**DOI:** 10.1007/s10670-025-00970-4

**Published:** 2025-06-19

**Authors:** Bahram Assadian

**Affiliations:** School of Philosophy, Religion and History of Science, University of Leeds, Woodhouse Lane, Leeds, LS2 9JT UK

## Abstract

A central thesis of neo-Fregean abstractionism is that numerical expressions of the form ‘the number of *F*s’, introduced by Hume’s Principle, should be read as genuine singular terms whose semantic function is to refer to particular objects. This paper explores the prospects of a variant of abstractionism in which such expressions have existential assertoric content, as in Russell’s analysis of definite descriptions. The neo-Russellian abstractionist faces three initial challenges: (i) the Russellian rendering of Hume’s Principle does not retain the ontological modesty that any admissible abstraction principle must respect. (ii) It defuses the objectual character of natural numbers, and thereby fails to ground their infinity. And (iii) it does not involve the means for engaging in identifying reference to numbers as particular objects, thereby constituting a derogation of arithmetical platonism. I shall investigate these challenges and propose solutions to address them.

## Abstractionism and Numerical Expressions

Abstractionism in the philosophy of mathematics is the thesis that Frege-style abstraction principles underwrite our knowledge of mathematical truths, the existence of mathematical objects, and our capacity to have singular thoughts about these objects. Abstraction principles are sentences of the following form:

$${\mathrm{(AP)}}\,\,\,\,\,\,\,\,\,\,\,\,\,\,\,\,\,\,\,\,\,\,\,\,\,\,\begin{aligned} \mathsection \alpha =\mathsection \beta \leftrightarrow \alpha \sim \beta \end{aligned}$$

where $\alpha$ and $\beta$ are variables of some type, $\mathsection$ is a term-forming operator that applies to such variables, and $\sim$ stands for an equivalence relation on the kinds of items over which the variables range. AP states that the abstract of $\alpha$ – i.e. the value of the abstraction function $\mathsection$ – is identical to the abstract of $\beta$ if and only if $\alpha$ and $\beta$ are related by $\sim$. (Here and below, I will omit the initial universal quantifiers $\forall \alpha$ and $\forall \beta$.) Any admissible instance of AP is an implicit definition of $\text {`}\mathsection$’ – where, in general, an implicit definition of an expression *e* consists of a sentence *S(e)* containing *e* and otherwise composed of already understood expressions. The idea is that we can fix the meaning of *e* as whatever it needs to be in order for *S*(*e*) to be true.

An important instance of an abstraction principle is known as Hume’s Principle, which states that the number of *F*s is identical to the number of *G*s if and only if there is a one-to-one correspondence between the *F*s and the *G*s. The neo-Fregean abstractionists formalize Hume’s Principle as follows:

$${\mathrm{(HP)}}\,\,\,\,\,\,\,\,\,\,\,\,\,\,\,\,\,\,\,\,\,\,\,\,\begin{aligned} \#F= \#G\leftrightarrow F\approx G \end{aligned}$$

where ‘ $F\approx G$’ is the second-order formalization of the claim that there is a relation *R* that one-to-one correlates the *F*s and the *G*s:

$$\begin{aligned} \exists R(\forall x(Fx\rightarrow \exists y\forall z((Gz\wedge Rxz)\leftrightarrow z=y))\wedge \forall x(Gx\rightarrow \exists y\forall z((Fz\wedge Rzx)\leftrightarrow z=y)))) \end{aligned}$$

HP purports not merely to fix the meaning of the numerical operator, and thereby the meaning of the associated singular terms, but also to provide epistemic access to what is represented by its left-hand side in terms of our antecedent knowledge of the matching right-hand sides.^1^

The neo-Fregean abstractionists – most notably, Hale and Wright (2001), Heck (2011a), and Linnebo (2018) – hold that the expressions formed on the left-hand side of HP are semantically singular terms. They have followed Frege who both in his *Die Grundlagen* *der Arithmetik* (Frege, 1884) and *Grundgesetze der Arithmetik* (Frege, 1903) takes the category of singular terms to include both semantically simple expressions, such as proper names and demonstrative pronouns, and semantically complex expressions including functional terms and definite descriptions. However, since the publication of Russell’s (1905) ‘On denoting’, there have been serious doubts as to whether the class of singular terms is as inclusive as Frege had proposed. According to the Russellian analysis of definite descriptions, part of what is asserted when we assert that, for instance, ‘The number of *F*s is even’ is that there is one and only one number of *F*s, which is even. When so construed, the left-hand side of Hume’s Principle states that there exists a unique object that numbers the *F*s, and there exists a unique object that numbers the *G*s, and whatever numbers the *F*s numbers the *G*s. Its right-hand side is the standard claim of one-to-one correspondence between the *F*s and the *G*s.

Let us, following MacFarlane (2009, p. 448), formalize the Russellian reading of Hume’s Principle as follows:

$${\mathrm{(HPR)}}\,\,\,\,\,\,\,\,\,\,\,\,\,\,\,\,\,\,\,\,\,\,\,\,\,\,\,\,\,\,\,\,\,\,\,\,\,\,\,\,\begin{aligned} (\exists !xNum(x,F) \wedge \exists !xNum(x,G) \wedge \forall x(Num(x,F) \leftrightarrow Num(x,G)))\leftrightarrow F\approx G \end{aligned}$$

where ‘*Num*(*x*, *F*)’ is interpreted as ‘*x* numbers the *F*s’, and ‘$\exists !xNum(x,F)$’ abbreviates the uniqueness of *x*: ‘$\exists x(Num(x,F)$ $\wedge$ $\forall y(Num(y,F)\rightarrow y=x)$’. There is, therefore, an important difference between HP and HPR: in the former, expressions of the form ‘the number of *F*s’ are introduced as singular terms whose semantic function is to refer to particular objects, whereas the latter treats such expressions along the Russellian semantics as a species of quantified expressions.

MacFarlane (2009, §1) asks whether it is essential for neo-Fregean abstractionism in taking ‘the number of *F*s’ to be a semantically singular term, as opposed to a Russellian quantified expression. Although in this paper, I shall critically examine some of Hale and Wright’s (2009, §1) rejoinders to MacFarlane, my primary aim is not to examine what would be lost from *neo-Fregean* abstractionism if we adopt Russellian semantics. The principal aim of this paper is to explore the prospects of a version of abstractionism, which I call *neo-Russellian abstractionism*, in which ‘the number of *F*s’ is introduced by HPR. The central thesis is that HPR has no distinctive disadvantages over HP when it comes to its credentials for the philosophical foundations of arithmetic.^2^

I investigate three initial challenges that the neo-Russellian abstractionist must face. The first concerns *ontological* *modesty*, which I discuss in Sect. 2. Any admissible abstraction principle must do no more than introduce the term which it purports to define; it must not, in particular, incorporate into the putative definition any further claim as to whether the newly defined term is non-empty. However,* prima facie*, HPR violates this constraint: since its left-hand side instances are existential claims, it appears to directly postulate the existence of numbers. I counter this challenge by showing that the ontological modesty of HP and that of HPR stands or falls together. There is, therefore, nothing distinctively problematic with HPR.^3^

The second challenge, which I discuss in Sect. 3, is directed at the Fregean thesis that numbers are objects. What is often held up as a key advantage of this thesis is that it sanctions Frege’s famous ‘bootstrapping’ argument for the existence of infinitely many natural numbers.^4^ However, since Fregean objects are what genuine (actual or possible) singular terms refer to, and since no such terms appear in HPR, it fails to preserve the status of numbers as objects, and thus fails to ground their infinity. It is correct to say that HPR, unlike HP, does not involve numerical singular terms purportedly referring to a range of objects; instead it features definite descriptions construed as quantifiers. However, as I argue in Sect. 3, the Fregean proof for the infinity of the natural numbers does not require the conception of objects as referents of *singular terms*. Instead, it rests on a less demanding conception of objects as values of *first-order variable*s. Nothing in HPR, I suggest, would preclude this latter conception.

The third challenge, addressed in Sect. 4, proceeds from the claim that HP, unlike HPR, specifies the truth-conditions of numerical identity sentences, which involve the most fundamental means for our *singular reference* to, or *identifying thoughts* about, numbers.^5^ The neo-Fregean abstractionists maintain that if it is not possible to convey singular reference to numbers as particular objects, the ‘platonist construal’ of arithmetical discourse must be given up. But would that come at a high cost for the neo-Russellian abstractionist? According to them, ‘the number of *F*s’, despite appearances to the contrary, is not to be understood as a genuine singular term referring to a particular object. In some sense, then, the neo-Russellian abstractionist must abandon the platonist, face-value reading of arithmetical discourse. This leads to a slight, though ultimately harmless, departure from platonism – or so the neo-Russellian suggests.^6^

What are the motivations for adopting the Russellian account of definite descriptions? In addition to the semantic, pragmatic, and inferential considerations that have led many philosophers and linguists to adopt Russellian semantics – powerfully defended by Neale (1990) – what I want to emphasize here, in view of the objectives of this paper, is that the Russellian treatment of definite descriptions can free us from the strong epistemic conditions often imposed on singular, object-involving thought. From a (neo-)Russellian perspective, ‘The number of *F*s is *G*’ expresses a proposition that is, in a sense, ‘about’ a particular object – its truth or falsity depends on how things stand with respect to the unique satisfier of the description, whatever it may be. However, the sentence merely expresses a general proposition whose truth-conditions do not involve what ‘the number of *F*s’, as a semantic unit, stands for. As a result, understanding ‘the number of *F*s’ does not require knowing *which* object uniquely satisfies the description: to understand the expression, it is not necessary to *identify* a particular object.

There is no doubt that providing a theory of singular thought and reference has proved to be a matter of considerable difficulty, especially in the case of abstract objects, which, if they exist, cannot enter into causal relations with us. Along this line, the neo-Russellian abstractionist accounts for the existence of numbers without requiring such a theory. In their view, ‘the number of *F*s’ stands for numbers, but it does not have the semantic function of effecting reference to a particular object, and thus cannot serve as the vehicle of object-involving thoughts. The question concerning the identification of numbers in our language and thought is thus misconceived. However, it is incumbent on the *neo-Fregean* abstractionist to provide a metasemantic account explaining the constitution of singular reference: what is it in virtue of which numerical singular terms refer to particular objects?

I believe that much more work needs to be done before my defense of neo-Russellian abstractionism against the challenges considered here could be said to be anywhere near complete. There are, moreover, other challenges that I have not addressed. I hope, however, that this paper indicates some promising directions for further inquiry.

## Ontological Modesty

One of the recurring themes of neo-Fregean abstractionism is that there is an epistemologically significant distinction between directly stipulating the existence of numbers by the Dedekind-Peano axioms, on the one hand, and deriving it from a meaning-constitutive stipulation in the form of HP, on the other. The neo-Fregean abstractionists thus aim to show how the existence of numbers rests on a principle which is ‘ontologically modest’, in that it does no more than fix the truth-conditions of identity sentences involving numerical terms on its left-hand side; it does not postulate the existence of numbers through a stipulation. This is manifested in the abstractionists’ canonical proof for the existence of numbers:

1. $$\begin{aligned} & \#F= \#F\leftrightarrow F\approx F \end{aligned}$$
2. $$\begin{aligned} & \quad F\approx F \end{aligned}$$
3. $$\begin{aligned} & \quad \#F= \#F \end{aligned}$$
4. $$\begin{aligned} & \quad \exists x(x= \#F) \end{aligned}$$

(1) is an instance of HP; (2) is the second-order logical truth that there are just as many *F*s as there are *F*s; (3) follows from (1) and (2); and (4) follows from (3) by Existential Introduction.^7^

The neo-Fregeans have always emphasized that the truth-value of the left- or the right-hand side of HP is no part of the stipulation. What is stipulated to be true, rather, is that the two sides of HP have the same truth-conditions. This explains why (3) does not rest on the logic of self-identity, but instead on the truth of (1) and (2) – that is, on the truth of HP and the satisfaction of the condition given on the right-hand side, which is independent from the stipulation of the truth of HP:

[A]n abstraction principle is a (universally quantified) *biconditional*, from which the existence of objects denoted by its instances’ left hand side terms follows *only* given the truth of the corresponding right hand sides. There is thus no question of any attempt simply to stipulate the existence of such objects. ((Hale, 2001), p. 343)

The existence of numbers thus follows only given the truth of the corresponding right-hand sides. To stipulate the truth of HP is not to stipulate the existence of numbers.

Let us assume, for the sake of argument, that the above line of reasoning establishes the ontological modesty of HP. It might be argued that even if HP sustains the required modesty, HPR doesn’t:

When so construed [i.e. numerical terms in terms of Russellian semantics], the left-hand side of an instance of Hume’s principle such as ‘The number of knives = the number of forks if and only if there are just as many knives as there are forks’ becomes ‘There is a unique number which belongs to the knives, and a unique number which belongs to the forks, and the former number is identical with the latter’. This rendering makes it patent how Hume’s principle postulates the existence of numbers, as well as explaining the concept of number. ((Rumfitt, 2003), p. 211, n. 35)

However, as in the case of any other abstraction principle, all that is stipulated by HPR is coincidence between the truth-values of the two sides of the biconditional. The left-hand side of HPR is indeed an existence claim, but that does not mean that HPR postulates the existence of numbers. The existence of numbers follows from HPR only given the truth of the co-ordinated sentence of the right-hand side. And the truth of the latter is independent of the stipulation. The following proof of numerical existence in terms of HPR substantiates this observation:

(5) $$\begin{aligned}  (\exists !xNum(x,F) \wedge \exists !xNum(x,F) \wedge \nonumber  \forall x(Num(x,F) \leftrightarrow Num(x,F)))\leftrightarrow F\approx F \end{aligned}$$
(6) $$\begin{aligned} & \quad F\approx F \end{aligned}$$
(7) $$\begin{aligned} & \quad \exists !xNum(x,F) \wedge \exists !xNum(x,F) \wedge \nonumber \forall x(Num(x,F) \leftrightarrow Num(x,F)) \end{aligned}$$
(8) $$\begin{aligned} & \quad \exists !xNum(x,F) \end{aligned}$$

The pattern of reasoning is precisely analogous to the (1)-(4) proof: (5) is an instance of HPR; (6) is a second-order logical truth. Since in (5), ‘$\exists !xNum(x,F)$’ occurs on the left-hand side of the biconditional, and is not being asserted outright, we need (6) in order to detach the left-hand side of (5), and infer (7). We can then use $\wedge$-elimination to establish (8). So, (6) plays the same role here as (2). In both cases, we do not directly postulate the existence of numbers from the initial stipulated abstraction principles; we need to assume the other supplementary premise. There is, therefore, no difference between HP and HPR – at least as far as postulating the existence of numbers is concerned.

Before moving on to the next section, let me address a potential concern about abstraction and ontological immodesty. If the existential claims featuring in HPR do not pose problems of ontological immodesty, why should we, within a Russellian framework, bother rephrasing the original identity statement at all? Why not simply stipulate that ‘$\exists !xNum(x,F)$’ is true if and only if *F* $\approx$ *F*? Why not say that there exists a unique object that numbers the *F*s just in case the *F*s are self-equinumerous? This would entail reformulating (5) as

(9) $$\begin{aligned} \exists !xNum(x,F) \leftrightarrow F\approx F \end{aligned}$$

and dropping (7). We will have the following argument for the existence of numbers:

(9) $$\exists !xNum(x,F)$$ $$\leftrightarrow F\approx F$$
(6) $$F\approx F$$
(8) $$\exists !xNum(x,F)$$

The existence of numbers would then follow only from (6) and (9) without the need for (5).

The mark of the modesty of HP is that the existence of numbers follows not from the direct stipulation of the truth of (3), but rather from the stipulated truth of (1) together with the truth of the corresponding right-hand sides. The same holds, *mutatis mutandis*, for the modesty of HPR. Given this conception of modesty, (9) is just as modest as (5), since (8) follows from (9) only if (6) is true.

 However, this, as such, does not warrant rejecting (5) in favor of (9). True, in terms of mere stipulation, (9) and (5) are equally modest. But in laying meaning-constitutive definitions, more than modesty is required. For instance, insofar as we are looking for a version of *abstractionism*, (9) does not qualify as an abstraction principle, as it does not conform to the general form of abstraction principles specified by AP:

$${\mathrm{(AP)}}\;\;\;\;\;\;\;\;\;\;\;\;\;\;\;\;\;\;\begin{aligned} \;\;\;\;\;\; \mathsection \alpha =\mathsection \beta \leftrightarrow \alpha \sim \beta \end{aligned}$$

One might rightly point out that (5) does not exhibit the general form of AP either. As MacFarlane (2009, p. 448) notes, though, if we write ‘#$F\text {'}$ as $\text {`}[$the $x$ $:Num(x,F)]\text {'}$, construed as a definite description, then HRP will be logically equivalent to the following principle, which *is* an instance of AP:

$$\begin{aligned} { {[} \text {the } x:Num(x,F)][ \text {the } y:Num(y,G)](x=y) \leftrightarrow F\approx G } \end{aligned}$$

The point is clearest when we consider the natural language rendering of the above principle, which closely mirrors the original Hume’s Principle: the number of *F*s is identical to the number of *G*s if and only if there is a one-to-one correspondence between the *F*s and the *G*s.

The second, related point against reasoning with (9) is that it does nothing more than specify the existence-conditions for numbers: no more is required for the existence of numbers than that of first-level properties and a second-level equivalence relation. However, as we noted in Sect. 1, the function of an abstraction principle is not only to fix the meaning of the abstraction function but also to provide epistemic access to the facts depicted by its left-hand side through our knowledge of the obtaining of the equivalence relation on its right-hand side. It is clear, though, that due to the limited resources of (9), any attempt to construct an abstractionist epistemology based on it will be futile.^8^

## Numbers as Objects

Part of the attraction of HP lies in its power for deriving the axioms of the Dedekind-Peano arithmetic. In particular, from HP, one can establish the existence of infinitely many natural numbers. In order to establish the existence part of this axiom, we must show that for every natural number *n*, there is a concept that is instantiated by *n*-many things, plus one more. The proof of this claim from HP requires making use of numbers themselves: we have to show that there is a concept instantiated by one more than zero thing; i.e. the concept of *being identical to 0*. This establishes the existence of 1. We can use this to establish the existence of 2, since the concept of *being a natural number less than or equal to 1* is instantiated by two things. The concept of *being a natural number less than or equal to 2* is instantiated by three things; and so on. That is the main idea behind Frege’s famous ‘bootstrapping’ proof for the existence of infinitely many natural numbers.^9^

It is often said – correctly, of course – that Frege’s proof would not go through if numbers were not *objects*. For instance, in their criticism of HPR, Hale and Wright write:

Given a broadly Fregean conception of objects, as *referents of actual or possible singular terms*, taking that surface syntax at face value means recognizing numbers as a kind of object. And of course the recognition of numbers as objects plays a crucial role in the execution of the programme. Most obviously, the proof – sketched by Frege in *Grundlagen* Sect. 82-3 – that every finite cardinal number is succeeded by another, presupposes that the numbers are objects, lying within the range of the* first-order quantifiers* implicit in Hume’s principle. A foundation along anything much like the lines just envisaged must at some stage provide for singular reference to numbers. Hume’s Principle does so right from the start, in the most direct way possible. ((Hale and Wright, 2009), p. 463; emphasis added)

But what does ‘object’ mean in the context of Frege’s logicist recovery of the axiom that every finite cardinal number is succeeded by another? Let us distinguish between two notions of object, both of which are mentioned in the above passage. According to the *quantificational* conception, to be an object is to be a value of a first-order variable. According to the *referential* conception, to be an object is to be a sort of thing that can be referred to by means of an actual or possible singular term. The latter characterization is often associated with Frege and the neo-Fregeans, and the former goes back to the work of Quine. Since the referent of a singular term can always serve as the value of a first-order bound variable, an object in the referential sense is also an object in the quantificational sense. The other direction, however, does not always hold: the legitimacy of quantification over a domain does not, as such, ensure that one can use singular terms to refer to the objects comprising that domain. An important example is the Russell-Quine method of eliminating (Fregean) singular terms and ordinary proper names in favour of quantified expressions.^10^

Frege’s bootstrapping proof requires numbers to have an objectual character only in the quantificational sense, and this much is respected by HPR. The proof essentially relies on the *impredicativity* of HP, in the sense that the singular terms on its left-hand side purport to refer to objects which are included in the range of the first-order quantifiers occurring on its right-hand side. (See the definition of $\text {`}F$ $\approx$ $G\text {'}$ in Sect. 1.) That is, the proof requires that the right-hand side of HP quantifies over numbers as falling under concepts whose numbers we consider. But HPR is also impredicative in the same sense: the definite descriptions occurring on its left-hand side purport to stand for objects which are included in the range of the first-order quantifiers featuring on its right-hand side. The impredicativity of HP and HPR does not depend on whether the expressions on their left-hand sides are genuine singular terms or quantified expressions, but rather on whether the objects these expressions stand for, if any, belong to a domain which is also the range of first-order variables occurring on the matching right-hand sides. In short, the impredicativity of HP and its Russellian analogue requires nothing beyond the quantificational conception of objects. Therefore, Frege’s proof for the infinity of natural numbers does not need anything more demanding than the quantificational conception.

Setting aside the bootstrapping proof, numbers, for Frege and the neo-Fregean abstractionists, may also serve as referents of genuine singular terms: they are objects also in the referential sense. Therefore, the expressions that are used to refer to them must have associated with them a class of sentences that settle the *criterion of identity *for these objects. This is part of what HP is supposed to do: not only is it a meaning-constitutive stipulation that fixes the meaning of the numerical operator, it also serves as the criterion of identity for numbers. So, to retain the objectual character of numbers in the Fregean, referential sense, one must have adopted suitable criteria not only for their numerical identity and distinctness, but also for their identification and re-identification.^11^ To understand ‘the number of *F*s’, construed as a genuine singular term, is to know (or truly believe) to *which* object it refers. For the neo-Russellian abstractionist, on the other hand, numbers are objects merely in the quantificational sense. To understand ‘the number of *F*s’ – construed as a Russellian definite description introduced by HPR – it is not necessary to know (or truly believe) which particular object uniquely satisfies it.

One might say that the above observation about the role of criteria of identity does not constitute a genuine difference between HP and HPR. For as stated in Sect. 1, the uniqueness clauses in HPR are formulated in terms of identity: ‘There is a unique number that numbers the *F*s’ is formulated as ‘$\exists x(Num(x,F)$ $\wedge$ $\forall y(Num(y,F)\rightarrow y=x)$’. How are we to understand the identity formulas if we lack criteria of identity to state that what is presented as such-and-such and what is presented as thus-and-so are indeed the same? It is true that understanding definite descriptions presupposes a capacity to understand identity. But that does not mean that our understanding of identity depends on a prior grasp of a *criterion of identity* – for two reasons.

First, as Hale (1987, p. 39) observes, not every judgment of identity involves an application of a criterion of identity. For instance, in the context of a game where one is asked to think of a number, I can tell you that the number of which I am thinking is 27, but that does not rest on my having applied any criterion of identity for numbers. The judgment that the number of which I am thinking is 27 is an identity judgment, but it is far from clear whether the terms flanking identity are genuine singular terms with which a criterion of identity is associated. Second, and more importantly, a criterion of identity must be distinguished from a *criterion of discernibility*. Someone might judge that 2 is not identical to 3, where this judgment is grounded, both metaphysically and epistemologically, in some other fact concerning their discernibility; e.g. 2 is the successor of 1; 3 is not. However, Frege and his followers would argue that this person does not yet possess a criterion of identity that can be used to identify and re-identify the referents of the associated singular terms on different occasions of use. According to them, HP not only provides a criterion of discernibility but also a criterion of identity that underwrites our identifying reference to numbers.^12^

Therefore, to restate the point, maintaining the objectual status of numbers in the Fregean sense requires adopting appropriate criteria not only for their numerical identity and distinctness but also for their identification. It should be clear that this cannot be sustained by the resources of HPR. In the next section, I explore some of the consequences of this claim for the neo-Russellian abstractionist.

## Singular Thought

There is no denying that the Russellian analysis of Hume’s Principle constitutes a radical departure from the standard neo-Fregean reading. The Russellian thought expressed by ‘The number of *F*s is *G*’ is that there is a unique object that numbers the *F*s and is *G*. Although ‘The number of *F*s is *G*’ is about a particular object – its truth or falsity depends on how things are with respect to a particular object – that does not entail that the sentence expresses a singular thought, or that the ingredient numerical expression is a genuine singular term. The vehicles of Russellian thoughts are purely descriptive; they are not directed at particular objects. Therefore, if we already have reasons in favour of our capacity for engaging in object-involving thoughts about numbers, HPR would clearly be of no use.

Indeed, this is what the neo-Fregean abstractionists take to be the main advantage of HP over HPR:

It seems to us, and we took it pretty well for granted, that people can and do engage in genuine *singular* *thought* about numbers, and that it ought, therefore, to be possible to introduce a range of terms [by HP] to serve as the primary vehicles for the expression of such thought. (Hale and Wright, 2009, p. 462)

I do not intend here to address the large question of whether we ‘can and do engage in genuine *singular thought* about numbers’ within the neo-Fregean framework. What I want to emphasize is that the neo-Russellian abstractionist would willingly sever the connection between what definite descriptions stand for and the alleged object-directedness of our thoughts. The neo-Russellian abstractionist would not be impressed by the neo-Fregeans’ claim that HPR must be dismissed on the grounds that it fails to provide us with means for singular thoughts about numbers. In fact, demanding an antecedent capacity to engage in such representations is precisely what the neo-Russellian abstractionist would reject: in her view, although we cannot direct our thoughts on numbers as particular objects, we often can ‘get at’ them, or talk about them, by describing them as the such-and-such or as the so-and-so. (To be more precise, even within a Russellian framework, there could be object-directed thoughts which are conveyed by ‘logically proper names’ – expressions whose content is simply what they refer to. Nevertheless, the category of Russellian logically proper names can be detached from neo-Russellian abstractionism. Following Quine (1948), the neo-Russellian may maintain that *all* singular terms are treated as quantified expressions.)^13^

But what would be lost if our numerical talk is not directed at numbers as particular objects by means of genuine singular terms? The neo-Fregeans respond as follows:

[T]he platonist construal of pure mathematical statements is utterly aimless unless it is possible for us to direct our thoughts into the individual objects which are taken to constitute those statements’ subject matter. (Wright (1983), p. 101)^14^

The ‘platonist construal’ of arithmetical sentences has weak and strong readings. The weak reading has it that an account of the truth-conditions of arithmetical sentences must accord with a referential, Tarski-style, semantics. We assign semantic values to sub-sentential expressions and then correlate the truth or falsity of a sentence with those semantic values. The semantic properties of sentences are thus determined in terms of relations (reference and satisfaction) between language and the world. This reading is weak since it does not preclude those semantic accounts which regard the surface syntax of arithmetical sentences as misleading with respect to their logical form. A classic example of such a semantic account is Russell’s theory of descriptions, which is adopted by our neo-Russellian abstractionist. According to this view, expressions of the form ‘the number of *F*s’, *despite appearances to the contrary*, are not to be understood as genuine names or singular terms referring to a range of objects.

It is utterly doubtful, though, whether such a weak requirement can live up to the platonist construal of arithmetical sentences that the neo-Fregean abstractionists recommend. The identifying numerical reference requires a reading of our arithmetical language, which – in addition to adopting a referential semantics – involves the demand to take it without further qualification that what *seem* to be singular terms *are* singular terms. If we press this strong requirement on the platonist construal of arithmetical sentences, then HPR will indeed constitute a derogation of arithmetical platonism.

But what will be lost from arithmetical platonism if we adopt neo-Russellian abstractionism? Platonism is often taken to be the thesis that mathematical objects *exist*; they are *abstract*; and they exist *independently* of our language and thought. In the rest of this section, I provide a very rough sketch of how the neo-Russellian abstractionist can uphold these central features of platonism.^15^

In Sect. 2, I have argued how HPR can be used to establish the existence of numbers, which are presumably abstract entities. Of course, we cannot simply assume that the expressions introduced by HRP stand for abstract objects – anymore than those introduced by HP. We need an argument here against what Heck (2011b) calls ‘semantic reductionism’, according to which names apparently of abstract objects, introduced by abstraction principles, do not refer to abstract objects, but to objects of some concrete sort. It would take me too far from my theme to explore this position in detail. But let me speculate that in the context of abstraction principles, the argument for abstractness does not hinge on whether the relevant objects are what genuine singular terms refer to (in the case of HP) or what definite descriptions stand for (in the case of HPR). That is, an account of the abstractness of objects does not depend on whether the notion of an object is to be understood in terms of the referential conception or the quantificational conception.

What about mind- or language-independence? An account of independence has not received enough attention in the abstractionist literature. The key gloss has been supplied in terms of the mind-independence of facts concerning the relation of one-to-one correlation or the purely general properties which stand in that relation: it is a matter of objective, mind-independent fact that there is a one-to-one correspondence between the *F*s and the *G*s, for the obtaining of such facts is entirely independent of the stipulation of the truth of HP. Since the existence of numbers is metaphysically grounded in facts concerning one-to-one correspondence, then, in so far as it is a mind-independent fact that there is a one-to-one correspondence between the *F*s and the *G*s, the existence of the number of *F*s and the number of *G*s is equally a mind-independent fact. If this is how an account of independence is to be given in light of HP, I cannot see how it could potentially fail to apply to HPR.^16^

Thus, HPR, just like HP, can accommodate the key elements of platonism: existence, abstractness, and independence. The gap between the forms of platonism underlying neo-Fregean and neo-Russellian abstractionism must be explained in terms of our (alleged) capacity to have *singular thoughts *about numbers: the thought one comes to understand by understanding HP, unlike HPR, inextricably involves an appreciation of the numerical expressions of the form ‘the number of *F*s’ as devices of genuine singular reference. I do not think that this departure from platonism will be of any serious concern to the neo-Russellian abstractionist.

## Footnotes

1 Various forms of abstractionism have been defended by Wright (1983), Hale and Wright (2001), Fine (2002), Heck (2011a), Rayo (2013), and Linnebo (2018).

2 In Assadian (2023), I explore the prospects of a version of abstractionism in which such numerical expressions have presuppositional content, as in Strawson’s account.

3 The ontological modesty of abstraction principles is one of the most recurring themes of neo-Fregean abstractionism. See, especially, Hale (2001) and Hale and Wright (2000, pp. 146–50).

4 See, for instance, Wright (1983, §xviii) and Dummett (1991, pp. 131–140).

5 See Wright (1983, §vii and xii), Hale (1987, ch. 7), Hale and Wright (2009), Heck (2011a, p. 14), and Linnebo (2005; 2018, ch. 8).

6 In some parts of Russell’s (1903) – for instance, §231 – Russell makes use of Fregean abstraction principles. Klement (2012, p. 149) discusses a Russellian version of Hume’s Principle, which states that the cardinal number of a class $\alpha$ is identical to the cardinal number of a class $\beta$ if and only if $\alpha$ and $\beta$ are equinumerous. As Klement points out, this is ‘almost identical’ to HP, but as he argues, given Russell’s ‘no-class’ theory, quantification over classes is to be eliminated in favour of quantification over propositional functions. The Russellian version of Hume’s Principle that is the focus of this paper differs from Klement’s, which aligns more closely with Russell’s own philosophy of mathematics. Hence, ‘neo-Russellian’ in the title of this paper. Jacinto (2024) develops another ‘neo-Russellian’ version of logicism in which cardinal numbers are conceived of as higher-order properties. However, according to the neo-Russellian who employs HPR, numbers are objects – not as referents of Fregean singular terms, but as values of first-order quantifiers. Moreover, Jacinto’s neo-Russellian logicism is not framed within a system of abstraction principles.

7 For more on this argument, see Wright (1983, pp. 147 and 159), Hale and Wright (2000, p. 146; 2001, pp. 309–310), and MacFarlane (2009, pp. 448–49). Some authors such as Tennant (1997) argue that the underlying logic of this argument must be a version of *free logic*. See Assadian (2024) for more discussion.

8 Linnebo (2018, pp. 59–60; ch. 12) factorizes abstraction principles into separate criteria of existence and of identity: where ABS(*u*, *x*) stands for $\text {`}x$ is the abstract of *u*’, the criterion of existence states that

$$\begin{aligned} u\sim u\rightarrow \exists x \text { Abs } (u,x) \end{aligned}$$

and the criterion of identity has it that

$$\begin{aligned} \text { Abs}(u,x) \wedge \text { Abs}(v,y) \rightarrow (x=y\leftrightarrow u\sim v). \end{aligned}$$

9 For the proof of the Dedekind-Peano axioms in second-order logic plus HP and other definitions, see Wright (1983, §xix), Boolos (1990), and Heck (2011a).

10 I owe the distinction between referential and quantificational conceptions of objects to Linnebo (2018, pp. 24–25), though the terminology is not his.

11  See Dummett (1981, pp. 73–80; 578–83) and Evans (1982) for an elaboration of this picture of singular reference. For the Evansian elements of neo-Fregean abstractionism, see Wright (1983, pp. 41–50; 101–3), Hale (1987, pp. 180–193; 264–265), and Linnebo (2005, p. 204; and 2018, ch. 8).

12 Thanks to an anonymous reviewer of this journal for prompting me to think about this question.

13 One may argue that if we render ‘the number of *F*s’ as ‘There exists an *x* such that *x* numbers the *F*s’, then to understand the latter, we must have an antecedent way to refer to numbers. For to understand and correctly use ‘*x* numbers the *F*s’, one must know a criterion that specifies what sort of objects it applies to, and under what conditions the objects of the relevant sort are identical or distinct – and this requires some antecedent understanding of what it would be for any identity sentence linking canonical numerical terms, *par excellence* terms of the form ‘the number of *F*s’, to be true. Therefore, ‘*x* numbers the *F*s’ cannot be understood without a prior understanding of ‘the number of *F*s’. The more general thesis underlying this argument is that to think of something as an *F* (where *F* is a sortal concept), one must know what it is to be an *F* and to know under what conditions the *F*s are distinguished from one another and also from non-*F* things. Discussing these (far from uncontroversial) requirements on singular thought, as defended by Evans (1982, ch. 4), would take us too far afield. For recent objections, see Burge (2010, pp. 171–99) and Goodman (2012).

14 For an extensive treatment of this observation, see Hale (1987, ch. 7).

15 See Linnebo (2023) for further discussion and other articles cited therein.

16 For more discussion on the neo-Fregeans’ take on independence, see Hale (1987, ch. 2.III and ch. 7.I), (Hale, 2021) and Linnebo (2018, 11.1).
